# Supplementary material for: High Fructose Intake Contributes to Elevated Diastolic Blood Pressure in Adolescent Girls: Results from The HELENA Study
Source: Nutrients. 2021 Oct 15;13(10):3608. doi: 10.3390/nu13103608 (PMC8538236; doi:10.3390/nu13103608)
Supplement: Supplementary file 1 [file nutrients-13-03608-s001.zip › nutrients-1358775-supplementary.pdf]

Supplementary Materials

Table S1 : Pure fructose and fructose total exposure intake content in various fructose-containing foods.

Dietary sources of fructose according to Mesana *et al* 2016 (39)	Pure Fructose (*per 100 g of food*)	Fructose total exposure* (*per 100 g of food*)	Reference
Sugar-sweetened beverages	4.9	5.4	Walker et al 2014 [44]
Non-chocolate confectionnary	2.6	22.6	Walker et al 2015 [45]
Chocolate	8.5	9.5	Sluik et al 2015 [46]
Cakes/pies/biscuits	3.5	15.8	Walker et al 2015 [45]
Desserts and puddings	2.1	5.1	Walker et al 2015 [45]
Breakfast cereals	1.2	11.4	Walker et al 2015 [45]
Others sources	2.7	2.7	Walker et al 2015 [45]
Fruit and vegetables juices	4.3	4.9	Walker et al 2015 [45]
Honey/Jam/Syrup	40.9	50.4	Raatz et al 2015 [47]
Fruits	7.0	8.5	Sluik et al 2015 [46]

*Fructose total exposure = Pure fructose (monosaccharide form) + fructose from sucrose (0.5 × sucrose per 100*g*)

Table S2. Data of confounding factors according to fructose exposure

	**Pure fructose**			**Total fructose exposure**		
	**Low**	**Middle**	**High**	**Low**	**Middle**	**High**
Number of adolescents	542	584	579	542	582	581
Gender (%boys)	33.0	44.4	63.2	33.4	43.3	63.9
Age (*years*)	14.6 ± 1.2	14.8 ± 1.2	14.9 ± 1.3	14.6 ± 1.2	14.7 ± 1.2	14.9 ± 1.3
Height (*cm*)	163.8 ± 8.3	165.7 ± 9.4	168.0 ± 9.0	163.3 ± 8.4	165.8 ± 9.0	168.3 ± 9.2
Body mass (*kg*)	56.5 ± 11.3	56.1 ± 11.6	58.3 ± 11.6	55.8 ± 10.6	56.6 ± 12.3	58.4 ± 11.5
Z-score BMI	0.28 ± 0.93	0.10 ± 0.85	0.14 ± 0.89	0.26 ± 0.91	0.13 ± 0.88	0.13 ± 0.88
MVPA (*min/d*)	47.6 ± 22.6	53.5 ± 26.7	61.0 ± 29.8	47.6 ± 22.7	53.1 ± 26.9	61.2 ± 29.4
Tobacco intake (%)	12.7	14.7	18.7	13.0	13.9	19.2
Salt intake (*mg/d*)	2309.8 ± 728.2	2407.8 ± 876.2	2684.2 ± 905.0	2308.2 ± 732.0	2410. ± 872.4	2682. ± 906.1
Energy intake (*kcal/d*)	2143.4 ± 445.6	2366.4 ± 496.1	2827.8 ± 711.3	2120.3 ± 416.3	2363 ± 498.7	2850.4 ± 702.6

Mean ± SD

Table S3. Comparison of main characteristics between the included and non-included adolescents

	**Included**	**Not included**	**P-value**	**Absolute standardized difference (%)**
Number of adolescents	1705	2024		
Gender (%boys)	52.8	51.8	0.53	4.3
Age (*years*)	14.7 ± 1.2	14.7 ± 1.2	0.48	9.0
Height (*cm*)	165.9 ± 9.1	165.8 ± 9.2	0.77	7.6
Body mass (*kg*)	57.0 ± 11.5	61.1 ± 13.4	**<0.001**	**26.6**
Z-score BMI	0.17 ± 0.89	0.56 ± 0.93	**<0.001**	**35.6**

Mean ± SD
